# Prognostic factors and predictive nomogram models for early death in elderly patients with hepatocellular carcinoma: a population-based study

**DOI:** 10.3389/fmolb.2023.1275791

**Published:** 2023-10-16

**Authors:** Hao Zhou, Junhong Chen, Kai Liu, Hongji Xu

**Affiliations:** ^1^ Department of Hepatobiliary and Pancreatic Surgery II, General Surgery Center, The First Hospital of Jilin University, Changchun, China; ^2^ Department of Abdominal Surgery, Guiqian International General Hospital, Guiyang, Guizhou, China

**Keywords:** hepatocellular carcinoma, elderly, early death, nomogram, SEER database

## Abstract

**Background:** Owing to an aging society, there has been an observed increase in the average age of patients diagnosed with hepatocellular carcinoma (HCC). Consequently, this study is centered on identifying the prognostic factors linked with early death among this elderly demographic diagnosed with HCC. Additionally, our focus extends to developing nomograms capable of predicting such outcomes.

**Methods:** The Surveillance, Epidemiology and End Results (SEER) database underpinned this study, showcasing participants aged 75 and above diagnosed with HCC within the timeframe from 2010 to 2015. These participants were divided randomly, at a 7:3 ratio, into training and validation cohorts. Univariable and multivariable logistic regressions were applied to the training cohort in the identification of prognostic indicators of early death, forming the basis for nomogram development. To measure the efficacy of these nomograms within both cohorts, we resorted to Receiver Operating Characteristic (ROC) curves, along with GiViTI calibration belt and Decision Curve Analysis (DCA).

**Results:** The study involved 1,163 elderly individuals diagnosed with HCC, having reported instances of 397 all-cause early deaths and 356 HCC-specific early deaths. The sample group was divided into two cohorts: a training group consisting of 815 individuals, and a validation cohort, comprised of 348 individuals. Multifactorial analysis identified grade, T-stage, surgery, radiation, chemotherapy, bone and lung metastasis as significant predictors of mortality from all causes. Meanwhile, race, grade, T-stage, surgery, radiation, chemotherapy, and bone metastasis were revealed to be estimative factors for cancer-specific mortality. Subsequently, these factors were used to develop nomograms for prediction. GiViTI calibration belt corroborated the acceptable coherence of the nomograms, DCA confirmed their valuable clinical applicability, and ROC curves evidenced satisfactory discriminative capacity within both training and validation cohorts.

**Conclusion:** The nomograms utilized in this study proved instrumental in detecting early death among elderly individuals afflicted with HCC. This tool could potentially assist physicians in formulating individualized treatment strategies.

## 1 Introduction

Hepatocellular carcinoma (HCC), a prevalent form of cancer globally, ranks third in mortality rates, contributing to over 780,000 deaths in 2018 as per the Global Cancer Statistics (Bray et al., 2018). Primary liver cancer encapsulates intrahepatic cholangiocarcinoma (ICC), HCC, among other types, with HCC constituting more than 80% of all liver cancer cases. Notably, HCC incidence has witnessed a surge in Europe and South America (Chu et al., 2019). Despite the advancements in treatment methods such as liver transplantation, surgery, chemotherapy, and radiation, the majority of HCC patients are diagnosed during the progressive phase, significantly impacting the prognosis (Forner et al., 2018).

Due to advancements in medical care, impressive socioeconomic growth, and an increase in average lifespan, aging is now being considered as a significant factor in the prognosis of many diseases, and we have observed a gradual increase in the average age of patients diagnosed with HCC (Yoon et al., 2013). Geriatric patients often exhibit a decline in both cardiopulmonary and liver regenerative functions, and a potential deterioration in their physical or nutritional status compared to younger demographic groups, which, unfortunately, could contribute to premature mortality (Sanz et al., 1999; Liang et al., 2013). The definition of “old age” varies, with ages such as 60, 70, 75, and 80 often being used as thresholds (Suda et al., 2013). Advanced age is recognized as an unfavorable prognosis factor in numerous types of cancer, including gastric, pancreatic (Lianyuan et al., 2021), breast (Huang et al., 2022), and colorectal (Yu and Zhang, 2020) cancers. It is important to note that, to our best knowledge, there have been limited targeted studies examining the prognosis of elderly patients with HCC.

The term “early death” pertains to survival within 3 months following a diagnosis (Kramer et al., 2007; Van et al., 2018; Janssens et al., 2020; Shen et al., 2021; Song et al., 2021). Comprehensive understanding of the relationship between prognostic factors of HCC and early death in older individuals with HCC could aid physicians in recognizing high-risk patients. This would further help in providing tailored treatment, considering the limited research available on this aspect.

Hence, in this research study, we acquired data of 1,163 elderly individuals diagnosed with HCC from the Surveillance, Epidemiology, and End Results (SEER) database to assess the link with prognostic factors. Furthermore, we developed and validated prediction nomograms for both all-cause and cancer-specific early deaths among older HCC patients. Such approaches have the potential to aid doctors in identifying high-risk individuals and establishing a personalized treatment strategy in a timely manner.

## 2 Materials and methods

### 2.1 Data source

Data for elderly patients (aged 75 years and above) with HCC used in this retrospective study were derived from the “Incidence SEER Research Plus Data 18 Registries, November 2020 Sub (2000–2018)” dataset within the SEER database, representing approximately 27.8% of American patients diagnosed with cancer. The SEER*Stat software (version 8.4.0, www.seer.cancer.gov) facilitated the extraction of these records. Given the public accessibility of the SEER database for research purposes, ethical committee approval was not required for this investigation.

**FIGURE 1:**
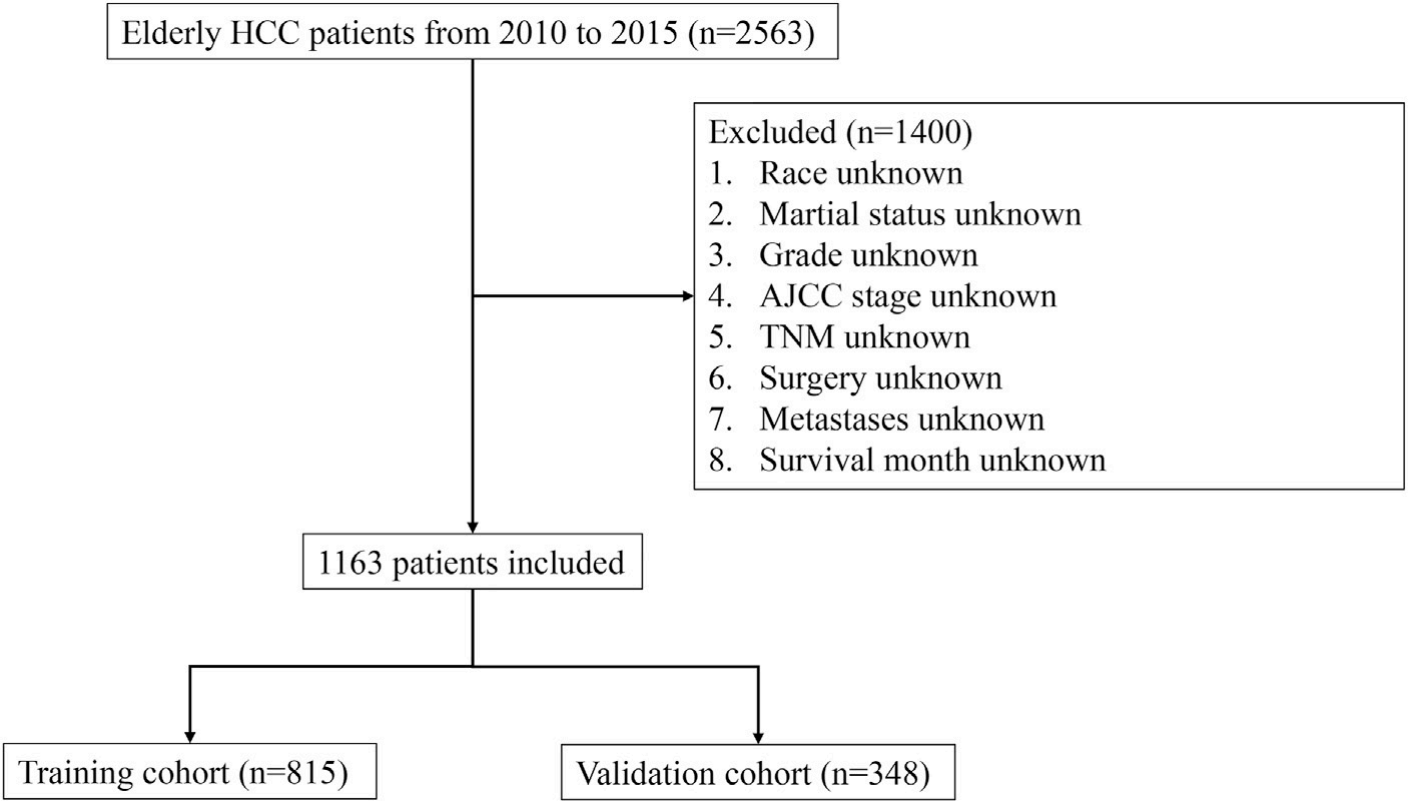
Flowchart illustrating the step-by-step process of patient selection from the Surveillance, Epidemiology, and End Results (SEER) database. The flowchart provides a visual representation of the inclusion and exclusion criteria, as well as the number of patients at each stage of the selection process.

The inclusion criteria for this study were as follows: 1) Histology codes based on the third version of the International Classification of Diseases for Oncology (ICD-O-3)—8170, 8171, 8172, 8173, 8174, and 8175; 2) Histological confirmation of HCC in patients; 3) Only cases where HCC was the primary tumor were studied; 4) Availability of complete demographic and medical information, including sex, race, marital status, grade, AJCC stage, T-stage, N-stage, M-stage, treatments (surgery, radiation, and chemotherapy), metastasis (bone, brain, lung), survival duration, cause of death, and vitality status. Early death, either from all-cause or specifically from cancer, was defined as mortality within 3 months of initial diagnosis. The research flowchart detailing these screening procedures is shown in Figure 1.

### 2.2 Statistical analysis

Quantitative data was represented using the mean and standard deviation (SD), whereas categorical variables were expressed in terms of number and percentage (n, %). A chi-squared test was used for comparing categorical data while a *t*-test served to evaluate quantitative data. This investigation randomly split participants into training and validation groups at a 7:3 ratio.

Prognostic factors for early death in HCC patients were established in the training group using both univariable and multivariable logistic regression techniques. We applied the “rms” package of the R programming language to produce predictive nomograms based on the prognostic factors determined by the multivariable logistic regression analysis. Factors that were statistically significant in the univariable logistic regression were further examined using multivariate logistic regression. To affirm the accuracy and reliability of the nomograms, we used Decision Curve Analysis (DCA), GiViTI calibration belt, and Receiver Operating Characteristic (ROC) curves in both the training and validation groups. We quantify the discriminative power of the nomograms using the area under the ROC curves (AUC): the closer the AUC to 1, the higher the discrimination. The clinical applicability of the nomograms was evaluated through DCA. All statistical analyses were performed with R programming language (version 4.0.0). A *p*-value less than 0.05 (two-sided) was considered the threshold for statistical significance across all analyses.

Our study eschewed traditional calibration assessment such as the Hosmer-Lemeshow statistical technique, due to its inherent limitation of exclusively examining the discrepancy between observed and forecast mortality rates sans indication of the direction, magnitude, or risk categories of these deviations. Additional common calibration assessment methodologies encompass the Hosmer-Lemeshow procedure amalgamated with classical calibration plots, notwithstanding, this method suffers from being reductive as it averages the risk amidst patients within each decile, thereby omitting comprehensive patient data. The more avant-garde GiViTI calibration belt statistical methodology has addressed these weaknesses, and it was deployed in our study to evaluate the calibration of all-cause early death and cancer-specific early death. The association between observed and expected results within the GiViTi calibration belt is ascertained by fitting a generalized polynomial logistic function between the logit transformation of forecast probabilities and the outcomes. The dark grey demarcations in Figure 4’s calibration belt denote the 95% CI boundaries, whereas the lighter grey interior symbolizes the 80% CI boundaries. A statistically significant bias can be deduced when the 95% CI boundaries of the GiViTI calibration belt do not incorporate the bisector, which represents the line of perfect calibration.

## 3 Results

### 3.1 Demographic and clinical characteristics

This study incorporated a sample of 1,163 elderly patients diagnosed with HCC. Among them, 356 cases resulted in early cancer-specific deaths, and a further 397 engendered instances of early deaths resulting from all causes. The demographic and pertinent data relating to the elderly HCC patients is presented in Table 1. The majority of the sample population were male (770, 66.2%) and white ethnicity (847, 72.8%). The demographic breakdown also indicates a marginally higher proportion of married patients (58%) compared to their unmarried counterparts (42%). The most common classification according to the AJCC was Stage I (526, 45.2%). The following in frequency were Stages II, III, and IV, which represent 13.6%, 28.3%, and 12.9% of the patient population respectively. In the context of cancer grading, Grade II was predominant among the elderly HCC sample relative to other grades. Lung metastases were the most frequently observed site of distant metastasis (64, 5.5%), followed by the bone (31, 2.7%), and the brain (3, 0.3%). A substantial proportion of patients underwent chemotherapy (400, 34.4%) and surgical interventions (281, 24.2%), while a lesser proportion received radiotherapy (91, 7.8%).

**TABLE 1:** Demographic and clinicopathological characteristics of elderly HCC patients in the SEER database.

	Characteristics	Number of patients			
		Overall (*n* = 1,163)	No early death (*n* = 766)	All-cause early death (*n* = 397)	Cancer-specific early death (*n* = 356)
Sex (%)	Male	770 (66.2)	501 (65.4)	269 (67.8)	241 (67.7)
	Female	393 (33.8)	265 (34.6)	128 (32.2)	115 (32.3)
Race (%)	White	847 (72.8)	549 (71.7)	298 (75.1)	269 (75.6)
	Black	79 (6.8)	47 (6.1)	32 (8.1)	28 (7.9)
	Other	237 (20.4)	170 (22.2)	67 (16.9)	59 (16.6)
Marital status (%)	Married	674 (58.0)	447 (58.4)	227 (57.2)	212 (59.6)
	Unmarried	489 (42.0)	319 (41.6)	170 (42.8)	144 (40.4)
Grade (%)	I	363 (31.2)	258 (33.7)	105 (26.4)	96 (27.0)
	II	532 (45.7)	357 (46.6)	175 (44.1)	153 (43.0)
	III	242 (20.8)	139 (18.1)	103 (25.9)	95 (26.7)
	IV	26 (2.2)	12 (1.6)	14 (3.5)	12 (3.4)
AJCC stage (%)	I	526 (45.2)	389 (50.8)	137 (34.5)	113 (31.7)
	II	158 (13.6)	125 (16.3)	33 (8.3)	28 (7.9)
	III	329 (28.3)	189 (24.7)	140 (35.3)	130 (36.5)
	IV	150 (12.9)	63 (8.2)	87 (21.9)	85 (23.9)
T (%)	T1	587 (50.5)	416 (54.3)	171 (43.1)	147 (41.3)
	T2	180 (15.5)	136 (17.8)	44 (11.1)	38 (10.7)
	T3	332 (28.5)	178 (23.2)	154 (38.8)	145 (40.7)
	T4	64 (5.5)	36 (4.7)	28 (7.1)	26 (7.3)
N (%)	N0	1,091 (93.8)	737 (96.2)	354 (89.2)	316 (88.8)
	N1	72 (6.2)	29 (3.8)	43 (10.8)	40 (11.2)
M (%)	M0	1,013 (87.1)	703 (91.8)	310 (78.1)	271 (76.1)
	M1	150 (12.9)	63 (8.2)	87 (21.9)	85 (23.9)
Surgery (%)	No	882 (75.8)	525 (68.5)	357 (89.9)	321 (90.2)
	Yes	281 (24.2)	241 (31.5)	40 (10.1)	35 (9.8)
Radiation (%)	No/Unknown	1,072 (92.2)	687 (89.7)	385 (97.0)	344 (96.6)
	Yes	91 (7.8)	79 (10.3)	12 (3.0)	12 (3.4)
Chemotherapy (%)	No/Unknown	763 (65.6)	432 (56.4)	331 (83.4)	298 (83.7)
	Yes	400 (34.4)	334 (43.6)	66 (16.6)	58 (16.3)
Bone metastasis (%)	No	1,132 (97.3)	756 (98.7)	376 (94.7)	336 (94.4)
	Yes	31 (2.7)	10 (1.3)	21 (5.3)	20 (5.6)
Brain metastasis (%)	No	1,160 (99.7)	766 (100.0)	394 (99.2)	353 (99.2)
	Yes	3 (0.3)	0 (0.0)	3 (0.8)	3 (0.8)
Lung metastasis (%)	No	1,099 (94.5)	743 (97.0)	356 (89.7)	316 (88.8)
	Yes	64 (5.5)	23 (3.0)	41 (10.3)	40 (11.2)

The study used a 7:3 allocation ratio to randomly assign participants into two main groups: a training group of 815 individuals, and a validation group of 348 participants. The demographic and clinical profiles of these geriatric HCC patients in both groups are detailed in Table 2. A comparison between the groups has not identified any statistically significant differences across key variables such as sex, race, marital status, grade, AJCC stage, T-stage, N-stage, M-stage, and treatment plan (surgical intervention, radiation therapy, chemotherapy). Additionally, the study assessed the occurrences of metastases in various organs—bone, brain, and lung. The corresponding *p*-values were 0.675, 0.775, 0.502, 0.784, 0.861, 0.787, 0.603, 0.791, 0.931, 0.762, 0.484, 0.48, 1, and 0.457.

**TABLE 2:** The baseline characteristics of the training and validation cohorts.

	Characteristics	Overall (*n* = 1,163)	Training cohort (*n* = 815)	Validation cohort (*n* = 348)	*p*
Sex (%)	Male	770 (66.2)	536 (65.8)	234 (67.2)	0.675
	Female	393 (33.8)	279 (34.2)	114 (32.8)	
Race (%)	White	847 (72.8)	593 (72.8)	254 (73.0)	0.775
	Black	79 (6.8)	53 (6.5)	26 (7.5)	
	Other	237 (20.4)	169 (20.7)	68 (19.5)	
Marital status (%)	Married	674 (58.0)	478 (58.7)	196 (56.3)	0.502
	Unmarried	489 (42.0)	337 (41.3)	152 (43.7)	
Grade (%)	I	363 (31.2)	261 (32.0)	102 (29.3)	0.784
	II	532 (45.7)	369 (45.3)	163 (46.8)	
	III	242 (20.8)	166 (20.4)	76 (21.8)	
	IV	26 (2.2)	19 (2.3)	7 (2.0)	
AJCC stage (%)	I	526 (45.2)	373 (45.8)	153 (44.0)	0.861
	II	158 (13.6)	110 (13.5)	48 (13.8)	
	III	329 (28.3)	225 (27.6)	104 (29.9)	
	IV	150 (12.9)	107 (13.1)	43 (12.4)	
T (%)	T1	587 (50.5)	419 (51.4)	168 (48.3)	0.787
	T2	180 (15.5)	124 (15.2)	56 (16.1)	
	T3	332 (28.5)	227 (27.9)	105 (30.2)	
	T4	64 (5.5)	45 (5.5)	19 (5.5)	
N (%)	N0	1,091 (93.8)	767 (94.1)	324 (93.1)	0.603
	N1	72 (6.2)	48 (5.9)	24 (6.9)	
M (%)	M0	1,013 (87.1)	708 (86.9)	305 (87.6)	0.791
	M1	150 (12.9)	107 (13.1)	43 (12.4)	
Surgery (%)	No	882 (75.8)	617 (75.7)	265 (76.1)	0.931
	Yes	281 (24.2)	198 (24.3)	83 (23.9)	
Radiation (%)	No/Unknown	1,072 (92.2)	753 (92.4)	319 (91.7)	0.762
	Yes	91 (7.8)	62 (7.6)	29 (8.3)	
Chemotherapy (%)	No/Unknown	763 (65.6)	529 (64.9)	234 (67.2)	0.484
	Yes	400 (34.4)	286 (35.1)	114 (32.8)	
Bone metastasis (%)	No	1,132 (97.3)	791 (97.1)	341 (98.0)	0.48
	Yes	31 (2.7)	24 (2.9)	7 (2.0)	
Brain metastasis (%)	No	1,160 (99.7)	813 (99.8)	347 (99.7)	1
	Yes	3 (0.3)	2 (0.2)	1 (0.3)	
Lung metastasis (%)	No	1,099 (94.5)	767 (94.1)	332 (95.4)	0.457
	Yes	64 (5.5)	48 (5.9)	16 (4.6)	

### 3.2 Prognostic factor analysis for early death

The study identifies prognostic factors associated with early death among geriatric HCC patients in a training group using univariable and multivariable logistic regression analysis. The results of this analysis are depicted in Tables 3, 4 respectively. Univariable logistic regression revealed a significant correlation between all-cause and cancer-specific early death, and factors such as race, grade, AJCC stage, T-stage, N-stage, M-stage, surgery, radiation, chemotherapy, bone metastasis, and lung metastasis. Multivariable logistic regression, accounting for the statistically significant features of the univariable analysis, confirmed grade, T-stage, surgery, radiation, chemotherapy, bone metastasis, and lung metastasis as prognostic factors for all-cause early death in geriatric HCC patients. The predictive factors for cancer-specific early death included race, grade, T-stage, surgery, radiation, chemotherapy, and bone metastasis.

**TABLE 3:** The univariable logistic regression analysis of all-cause and cancer-specific early death in elderly HCC patients.

Characteristics	All-cause early death			Cancer-specific early death		
	OR	95% CI	*p*	OR	95% CI	*p*
Sex						
Male	Ref			Ref		
Female	0.82	0.6–1.11	0.203	0.86	0.63–1.18	0.343
Race						
White	Ref			Ref		
Black	1.15	0.65–2.05	0.626	1.13	0.63–2.04	0.679
Other	0.64	0.44–0.93	0.02	0.61	0.41–0.9	0.014
Marital status						
Married	Ref			Ref		
Unmarried	0.91	0.68–1.22	0.53	0.81	0.6–1.09	0.166
Grade						
I	Ref			Ref		
II	1.1	0.78–1.54	0.598	1.06	0.75–1.52	0.73
III	1.76	1.18–2.65	0.006	1.86	1.23–2.8	0.003
IV	3.17	1.23–8.18	0.017	2.97	1.16–7.62	0.023
AJCC stage						
I	Ref			Ref		
II	0.61	0.35–1.04	0.068	0.6	0.34–1.08	0.091
III	1.99	1.41–2.83	<0.001	2.41	1.68–3.46	<0.001
IV	4.39	2.8–6.91	<0.001	5.49	3.47–8.69	<0.001
T						
T1	Ref			Ref		
T2	0.61	0.38–0.99	0.044	0.61	0.37–1.02	0.06
T3	2.09	1.49–2.91	<0.001	2.36	1.68–3.32	<0.001
T4	1.84	0.99–3.43	0.056	2.28	1.22–4.26	0.01
N						
N0	Ref			Ref		
N1	2.6	1.44–4.68	0.002	2.55	1.42–4.59	0.002
M						
M0	Ref			Ref		
M1	3.69	2.42–5.63	<0.001	4.25	2.79–6.48	<0.001
Surgery						
No	Ref			Ref		
Yes	0.21	0.14–0.33	<0.001	0.22	0.14–0.35	<0.001
Radiation						
No/Unknown	Ref			Ref		
Yes	0.43	0.23–0.83	0.011	0.51	0.26–0.97	0.04
Chemotherapy						
No/Unknown	Ref			Ref		
Yes	0.25	0.17–0.35	<0.001	0.25	0.17–0.36	<0.001
Bone metastasis						
No	Ref			Ref		
Yes	6.02	2.36–15.35	<0.001	5.68	2.32–13.87	<0.001
Brain metastasis						
No	Ref			Ref		
Yes	NA	NA	0.97	NA	NA	0.97
Lung metastasis						
No	Ref			Ref		
Yes	4.16	2.24–7.73	<0.001	4.91	2.64–9.13	<0.001

**TABLE 4:** The multivariate logistic regression analysis of all-cause and cancer-specific early death in elderly HCC patients.

	All-cause early death			Cancer-specific early death		
	OR	95% CI	*p*	OR	95% CI	*p*
Race						
White	Ref			Ref		
Black	NA	NA	NA	0.84	0.42–1.69	0.633
Other	NA	NA	NA	0.61	0.38–0.98	0.042
Grade						
I	Ref			Ref		
II	1.11	0.74–1.67	0.625	1.01	0.66–1.54	0.962
III	1.88	1.17–3.03	0.01	1.97	1.21–3.2	0.006
IV	2.05	0.67–6.34	0.21	1.77	0.58–5.38	0.311
T						
T1	Ref			Ref		
T2	0.87	0.5–1.51	0.63	0.9	0.5–1.63	0.737
T3	2.35	1.57–3.51	<0.001	2.83	1.87–4.28	<0.001
T4	1.92	0.9–4.08	0.09	2.6	1.23–5.51	0.013
N						
N0	Ref			Ref		
N1	1.77	0.87–3.59	0.112	NA	NA	NA
M						
M0	Ref			Ref		
M1	NA	NA	NA	1.79	0.83–3.84	0.135
Surgery						
No	Ref			Ref		
Yes	0.16	0.1–0.26	<0.001	0.19	0.11–0.32	<0.001
Radiation						
No/Unknown	Ref			Ref		
Yes	0.18	0.08–0.41	<0.001	0.23	0.1–0.5	<0.001
Chemotherapy						
No/Unknown	Ref			Ref		
Yes	0.14	0.09–0.21	<0.001	0.14	0.09–0.22	<0.001
Bone metastasis						
No	Ref			Ref		
Yes	18.12	4.86–67.59	<0.001	8.71	2.09–36.29	0.003
Lung metastasis						
No	Ref			Ref		
Yes	2.72	1.32–5.59	0.007	2.16	0.79–5.9	0.132

### 3.3 Nomogram construction

**FIGURE 2:**
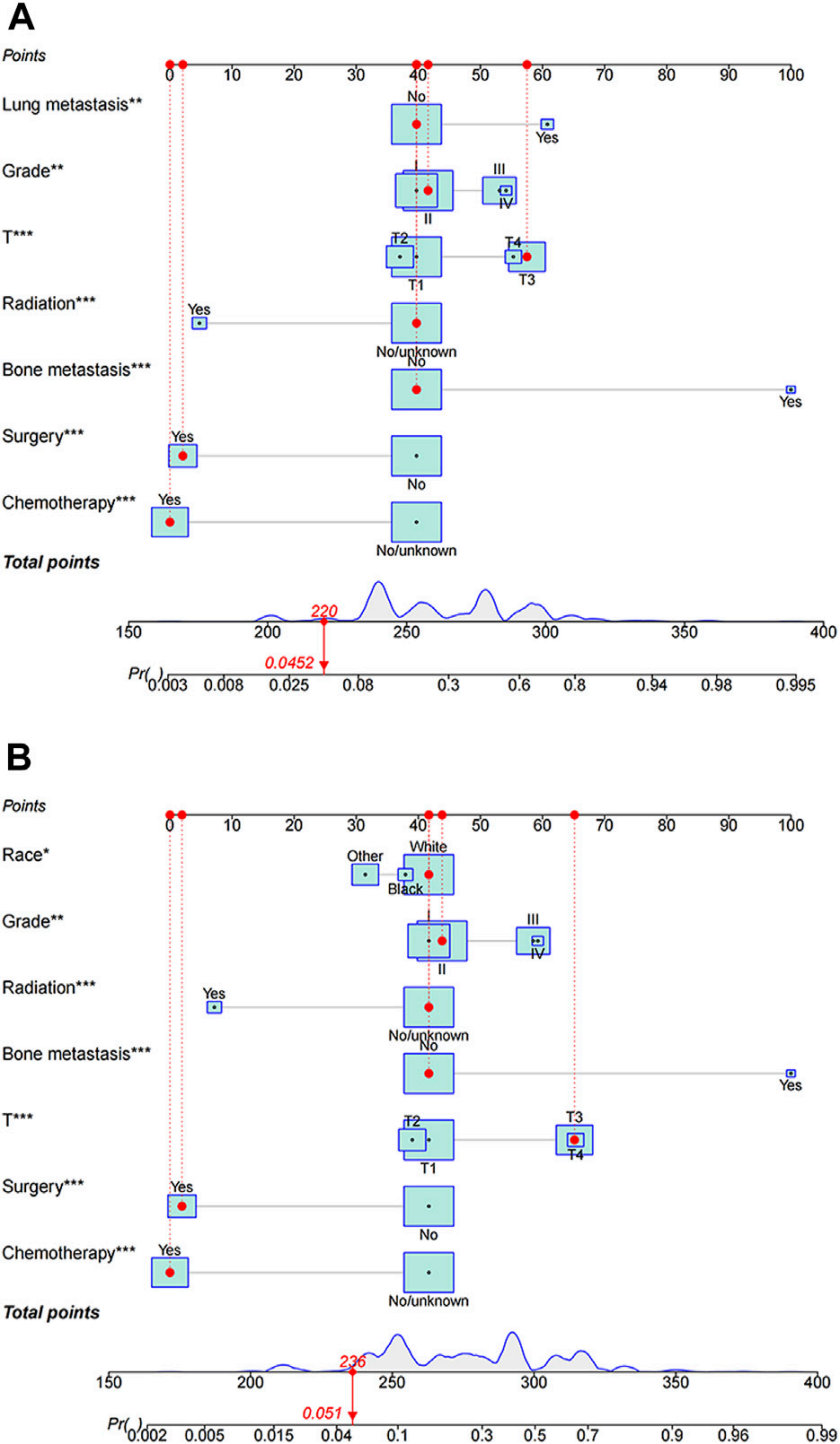
Nomograms predicting all-cause **(A)** and cancer-specific early death **(B)** in elderly patients with hepatocellular carcinoma (HCC). All-cause early death and cancer-specific early death refer to mortality within 3 months of initial diagnosis, whether due to any cause or specifically due to HCC, respectively.

The study used multivariate logistic analysis to identify significant prognostic factors linked to all-cause and cancer-specific early death in elderly individuals with HCC. These factors were integral in the development of predictive nomograms, a valuable tool in this area of research. Figure 2A visually represents these influential factors, emphasizing bone metastasis as the strongest predictor of all-cause early death in this population. Similarly, Figure 2B underscores the significant role of bone metastasis in determining the likelihood of early cancer-specific death.

Within the framework of these predictive nomograms, every determinant is ascribed specific points. The total score is produced by integrating all of the scores attributed to the different variables. This total score subsequently reveals the respective probability of the occurrence of premature death in these patients. The constructed model is instrumental in identifying population-based probabilities of negative outcomes. It enables the customization of individual patient management by taking into account the severity of their condition and the related variables.

For instance, consider an elderly patient diagnosed with a grade IV, T4 category cancer, having lung metastasis and having only received surgical intervention. In this scenario, the predictive nomogram indicates an associated all-cause early death probability verging on 58%. Conversely, in another hypothetical case where a patient, despite carrying a diagnosis of Grade IV and T4 cancer and having received only surgery as a treatment modality, yields an estimated lower cancer-specific early death probability, closely approximating 30%. This discrepancy underscores the significance of the interplay of numerous factors in determining the projected outcomes, further emphasizing the need for comprehensive consideration of multiple patient-specific factors when strategizing management plans.

### 3.4 Performance and validation of nomograms

**FIGURE 3:**
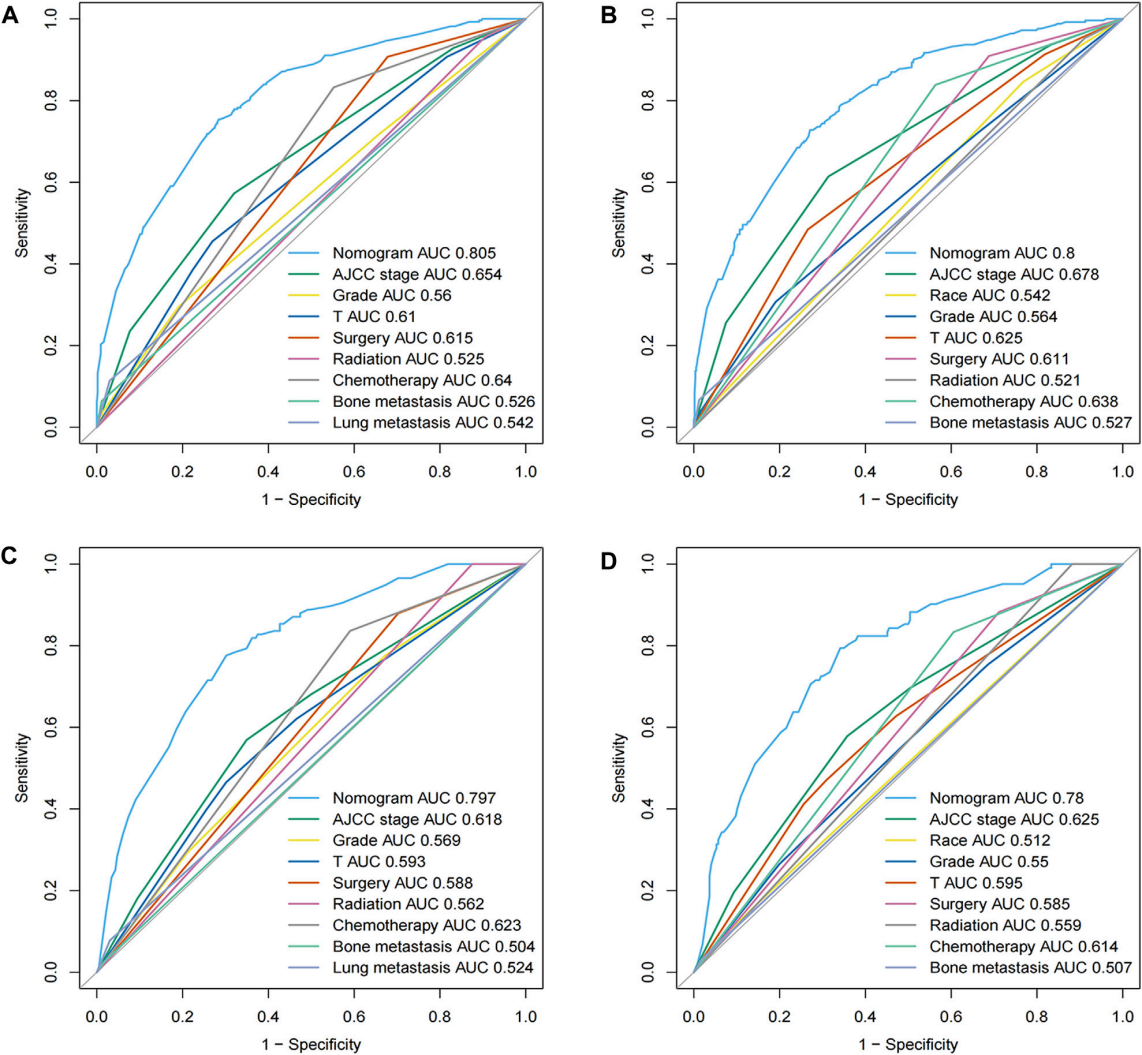
ROC curves for the nomograms in panels **(A–D)** depict the efficacy of our predictive models for all-cause and cancer-specific early death. **(A,B)** represent the training cohort, while **(C,D)** illustrate the validation cohort. The ROC curve provides a graphical representation of the true positive rate against the false positive rate, offering insights into the accuracy and discriminative ability of the nomograms.

The comparison of ROC curves across the evaluated cohorts uncovered a prominent performance superiority in AUC values generated from the nomograms, significantly exceeding those obtained from individual parameters. Specifically, the all-cause early death nomogram exhibited an impressive discriminative capacity with an AUC of 0.805 and 0.797 in the training and validation cohorts, respectively, effectively overshadowing the AUC value of 0.654 and 0.618 obtained from the conventional AJCC staging (Figures 3A, C). Notably, these outcomes remain invariable, regardless of the chosen nomogram or AJCC staging, persistently surpassing the AUC values linked to independent predictors such as grade, T-stage, surgery, radiation, bone and lung metastasis. Further, extending this analysis to the cancer-specific early death nomogram yielded similar results with an AUC value of 0.8 and 0.78 for the training and validation cohorts, respectively. This could be interpreted as strong evidence of the broad-ranging discriminatory power of the nomogram, that again supersedes the AUC value of 0.678 and 0.625 derived from the traditional AJCC staging (Figures 3B, D). This outcome maintains a consistent pattern in outperforming the AUC values associated with independent predictors including race, grade, T-stage, bone metastasis and different treatment modalities. The approximate closeness of these AUC values to the good discriminative value of 0.9, as graphically depicted in Figures 3A–D, reaffirms the superior robust discriminative proficiency encompassed in these nomograms. With these nomograms, it is clear that the path to enhance diagnostic prediction and therapeutic decision-making outcome could be significantly improved.

**FIGURE 4:**
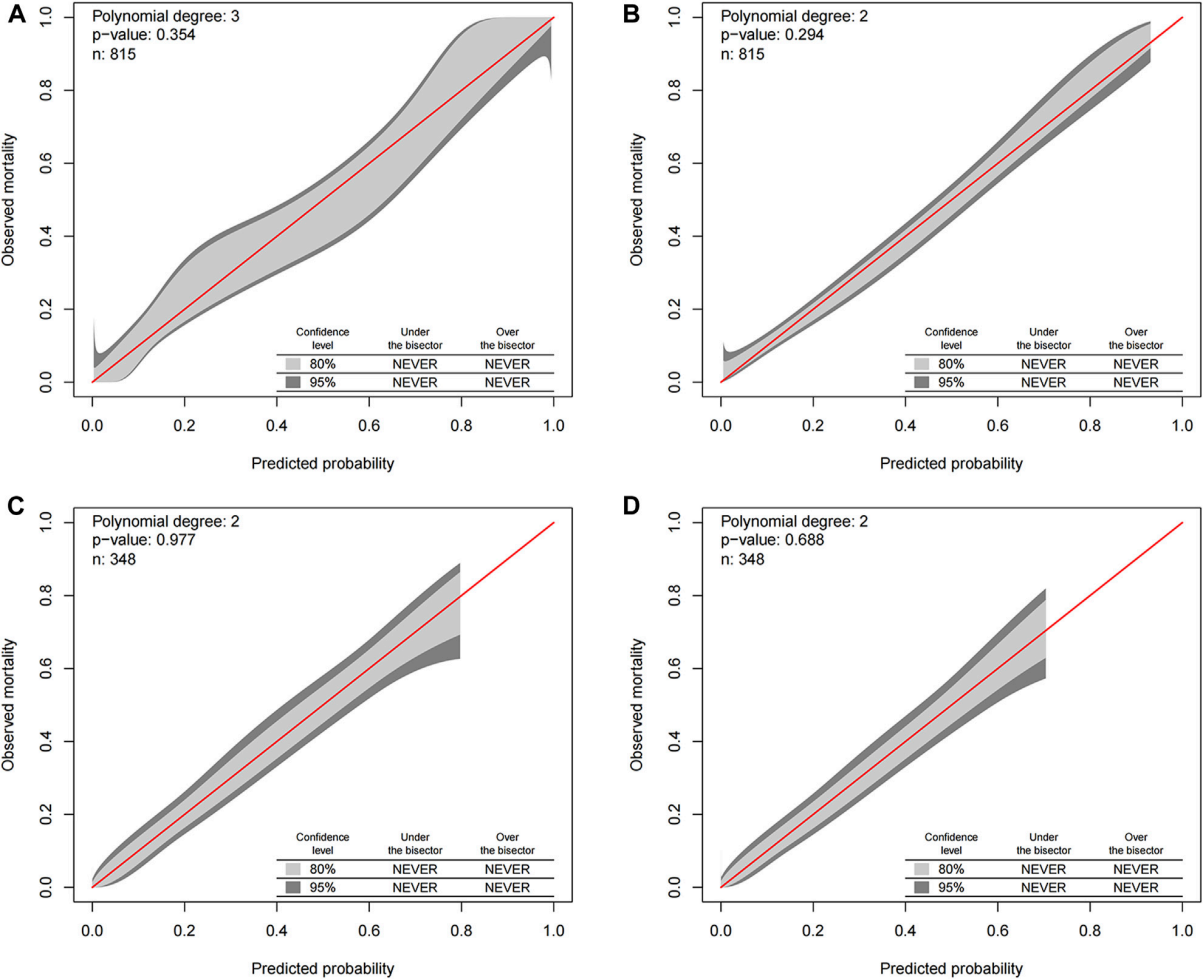
GiViTI calibration belt for predicting all-cause and cancer-specific early death in the training **(A,B)** and validation **(C,D)** cohorts. The GiViTI calibration belt visually represents the calibration of the predictive model. The dark grey demarcations within the belt indicate the 95% confidence interval (CI) boundaries, while the lighter grey interior represents the 80% CI boundaries. The bisector line, which signifies the line of perfect calibration, should ideally be encompassed within the 95% CI boundaries of the calibration belt. A statistically significant bias in the model’s predictions can be inferred if this bisector is outside the 95% CI boundaries.

For our assessment, we included all data collated from both the training and validation sets, embodying all-cause early death and cancer-specific early death, presenting overall consistent patterns between the predicted and actual values. The bisector in Figure 4 is positioned within the 95% CI dark grey segment of the GiViTI calibration belt, visibly displaying no deviation and no statistically significant bias, indicative of satisfactory calibration. This correspondingly reflects a sturdily congruous alignment between the projected probabilities and recorded early mortalities, thereby bolstering the validity of the predictive model applied. The robust consistency between the hypothesized and observed outcomes proffers compelling substantiation for optimal calibration, adding robust credibility to our results and conclusions.

**FIGURE 5:**
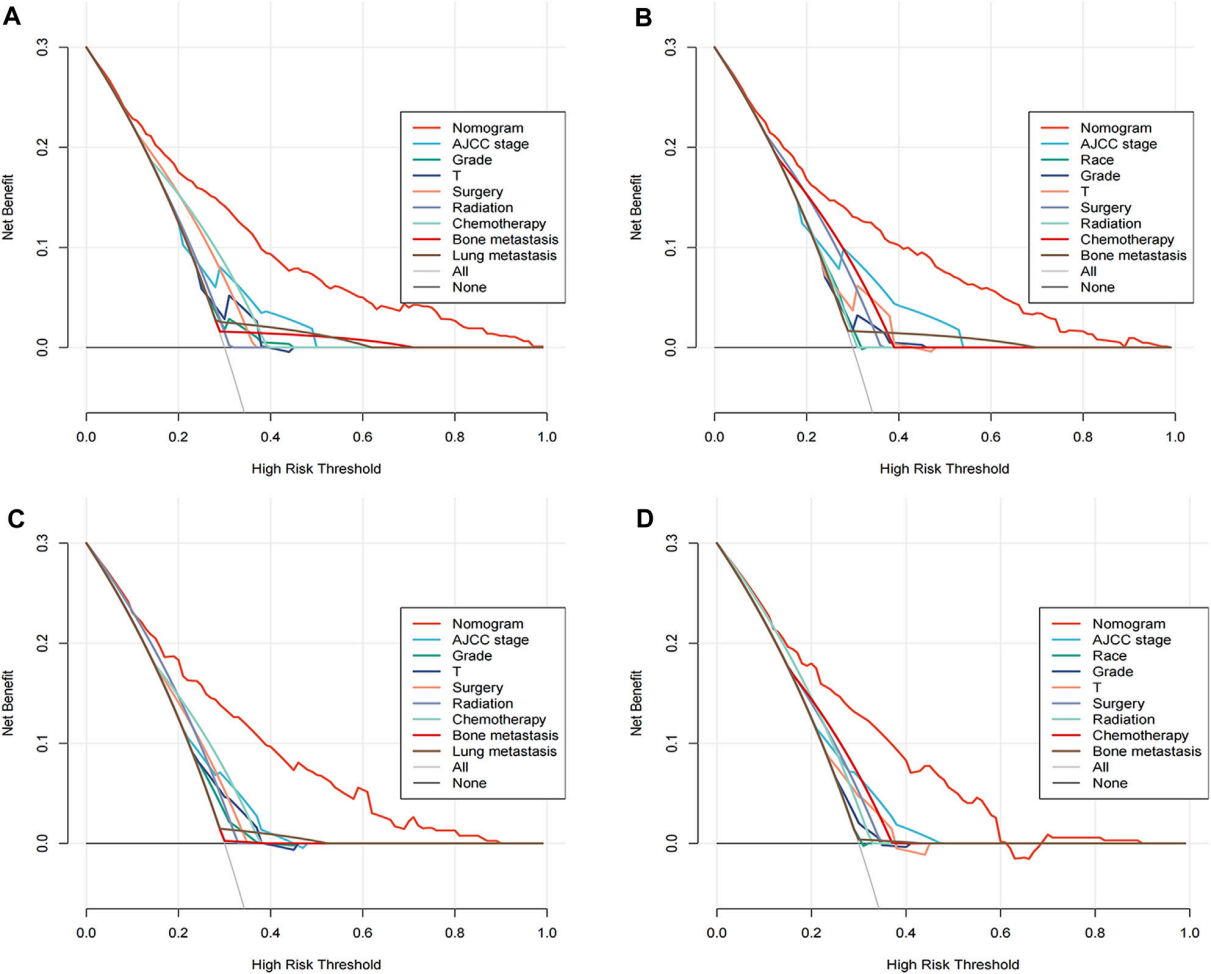
Decision curve analysis (DCA) illustrating the clinical utility of the nomograms. DCA evaluates the net benefit of using the nomograms to predict all-cause and cancer-specific early death. The training cohorts are represented in panels **(A)** for all-cause and **(B)** for cancer-specific predictions, while the validation cohorts are shown in panels **(C,D)** respectively.

A comprehensive appraisal was executed to ascertain the clinical applicability of the nomograms, employing the DCA curves. The ensuing analysis unveiled a significant manifestation, internalizing the conglomerated efficacy of these nomograms conspicuously surpassed the individual variables when examined in isolation. This augments the pivotal standpoint of the nomograms in terms of their superior capacity in the interplay of predictors, lay bare in an analytical format, thereby assisting in better decision-making. Asserting the dominance of the nomograms in prediction becomes more palpable in Figure 5, where it diligently delineates the real-world implications of these nomograms. The figure explicitly illustrates the multifarious advantages offered by the nomograms for foretelling both the all-cause and cancer-specific early death in patient cohorts, crucially in both the training and validation sets.

Cumulatively, amalgamating the findings from the ROC curves, GiViTI calibration belt, and the DCA analyses offers unassailable validation for the effectiveness of the proposed nomograms in predictions. These various analyses unequivocally corroborate the superior performance of the nomograms over standalone variables in both accurately and efficiently predicting early death incidents, thus emphasizing their substantial potential for clinical implementation and utility.

## 4 Discussion

Recent advancements in HCC treatment, which include liver transplantation, radiofrequency ablation, targeted therapy, and immunotherapy, have increased the lifespan of HCC patients despite the persistently high morbidity and mortality rates (Xie et al., 2020). Moreover, HCC mortality rates have seen a significant rise in Western Europe over the past four decades (Ascione et al., 2017). Despite this, few studies have explored early HCC patient mortality. With improvements in healthcare, socioeconomic development, and rising life expectancy, HCC incidence has surged, and the prognosis for geriatric HCC patients remains a global challenge (Balducci and Extermann, 2000). Aging correlates with increased disability rates, progressive functional reserve depletion in multiple organ systems, and decreased tolerance to various stresses, including social, emotional, and physical ones. Consequently, old age has been identified as a poor prognostic factor for various cancers, including lung (Tas et al., 2013), thyroid (Haymart, 2009), prostate (Bechis et al., 2011), and HCC (Guo et al., 2017). Therefore, to provide personalized treatment plans for elderly patients and acknowledge those at risk of early death due to HCC, more focus should be given to this population to identify risk factors associated with early HCC death.

In a comprehensive study analyzing patients with advanced HCC, researchers identified certain attributes as significant risk factors. These included, but were not limited to, age, histological grade, tumor stage (T-stage), surgical procedures, engagement with radiotherapy and chemotherapy, and the occurrence of metastasis to the bones and lungs (Zhang et al., 2022). To the best of our knowledge, such a exploration focusing on the discovery of prognostic indicators for HCC in geriatric patients, and further developing nomograms for predicting potential early death in these patients, represents an unprecedented approach in this field. Upon further breakdown of the study’s results, it was found that 397 out of 1,163 subjects, constituting 34.14% of those included in the study, suffered from an early death due to all foreseeable causes. Additionally, 30.61% of the patients (equivalent to 356 out of 1,163 subjects) succumbed specifically to cancer-related causes. These results underscore the lethal potency of HCC in the elderly, where patients primarily die due to the malignancy itself rather than other concurrent health conditions. Despite the empirical observation that HCC most frequently develops in males, a revelation of the study was the absence of any appreciable gender influence on the prognosis of HCC patients (Golabi et al., 2017). In order to better comprehend the risk of early death among these patients, the study took into account the histological grade, tumor stage (T3-T4), non-receipt of surgical treatment or non-participation in radiotherapy and chemotherapy, and presence of bone and lung metastasis. The complex interplay of these factors significantly escalated the risk of an early demise in the subjects, particularly those suffering from advanced HCC (Chen et al., 2019; Zhang et al., 2019; Zhang et al., 2021). In essence, this study determined that elderly patients with a higher histological grade and stage T3 or T4, and who did not undergo surgery, radiotherapy or chemotherapy for their condition, along with those who have bone and lung metastasis, are severely predisposed to early death caused by HCC. In addition to this, racial disparities (namely, among white and black patients), a higher tumor grade, T3 or T4 stage, absence of surgical treatment, non-participation in radiotherapy or chemotherapy, and presence of bone metastasis all predicted a heightened risk of early death specifically related to cancer.

In the management of cancer in elderly patients, it is notable to take into account their increased susceptibility to postoperative complications compared to younger patients. This predisposition often results in a delayed initiation of subsequent oncological interventions such as radiotherapy and chemotherapy, thereby diminishing the resultant survival benefits for this particular demographic (Liang et al., 2013; Yang et al., 2022). HCC is recognized as an aggressive form of carcinoma, with a significant proportion of patients (14.0%–36.7%) presenting with extrahepatic metastases (Natsuizaka et al., 2005). In the context of this study, it was established that the lung, with a recorded incidence rate of 5.5%, represented the predominant site of extrahepatic metastasis. Other identified metastatic sites included the bone (2.7%) and brain (0.3%). Despite these findings, instances of HCC with brain metastasis were exceptionally low within the confines of this study, with only three reported cases. As such, these findings must be treated tentatively, underscoring the importance of conducting more extensive, large-scale prospective studies before this can be conclusively verified.

The nomogram, a straightforward and easily accessible instrument, has found extensive application in malignant tumor prognostic models. It not only allows for a comprehensive risk and prognosis assessment of carcinomas (Yan et al., 2021), but also aids in tailoring personalized clinical decisions and optimizing cancer treatments (Balachandran et al., 2015). In this particular study, we utilized the SEER database, a comprehensive source of high-quality, large-scale data, to construct the nomograms. This resource guaranteed the stability and reliability of our results (Shen et al., 2021). Recent research by Song et al. (2020) demonstrated that the nomogram derived from the SEER database outperformed the Federation of Gynecology and Obstetrics (FIGO) staging system in the prediction of early death of uterine sarcoma. Similarly, Yang et al. (2020) concluded that the nomogram produced an AUC of 0.847 in predicting the early mortality of patients diagnosed with advanced gastric cancer. In the scope of this research, we discovered that the AUCs of nomograms predicting all-cause and cancer-specific early death were significantly higher than those of single variables in both the validation and training groups, thereby underscoring the nomograms’ strong predictive ability. Calibration curve analysis was used to compare expected and observed outcomes (Vickers et al., 2016), further supporting the successful performance of the nomograms. The DCA curves pointed to a significant correlation between the predicted efficiency of nomograms and their actual clinical application in this research. Thus, the nomograms constructed in this study hold potential as predictive models that could assist clinicians in designing clinical studies, identifying personalized therapy options, and adjusting follow-up strategies.

It is important to acknowledge the constraints and limitations inherent in this investigation. Primarily, this study is retrospective in nature which inherently introduces inevitable selection bias in the samples studied. Consequently, caution is warranted in the interpretation of the findings. Secondly, while the findings indicated robust predictive capabilities rendered by the nomograms, it is crucial to note that our data source was the SEER database, a database indigenous to the US. This potentially limits the generalizability of the results. Therefore, in order to enhance the validity and applicability of the findings, additional data from a broader sample size and multiple global research centers would be instrumental in refining these results and providing a holistic view. Lastly, when considering the early death prognosis of geriatric subjects presented with HCC, it should be noted that there are other potential risk factors at play. It is crucial to factor in adverse living habits, specific treatment interventions implemented, the existing medical history of the patient, and the presence of tumor markers, all of which might significantly contribute and influence the study’s outcomes. Hence, future investigations must broaden their scope and consider these variables, in order to comprehensively understand the phenomenon under study.

## 5 Conclusion

Drawing from the SEER database, this study identified various independent risk factors associated with all-cause and cancer-specific early death in patients diagnosed with HCC. Utilizing the discerned risk factors, two predictive nomograms were subsequently constructed and subjected to rigorous validation processes. The resulting models demonstrated satisfactory performance, leading us to conclude that these nomograms could potentially have profound clinical implications. Indeed, their application could substantially assist healthcare practitioners in designing comprehensive clinical studies, determining personalized treatment strategies, and modifying follow-up protocols. Collectively, these three factors have the potential to enhance the survival outcomes of patients diagnosed with HCC significantly.

## Data Availability

The original contributions presented in the study are included in the article/Supplementary Material, further inquiries can be directed to the corresponding author.
